# 5qSMA: standardised retrospective natural history assessment in 268 patients with four copies of *SMN2*

**DOI:** 10.1007/s00415-024-12188-5

**Published:** 2024-02-27

**Authors:** Katharina Vill, Moritz Tacke, Anna König, Matthias Baumann, Manuela Baumgartner, Meike Steinbach, Guenther Bernert, Astrid Blaschek, Marcus Deschauer, Marina Flotats-Bastardas, Johannes Friese, Susanne Goldbach, Martin Gross, René Günther, Andreas Hahn, Tim Hagenacker, Erwin Hauser, Veronka Horber, Sabine Illsinger, Jessika Johannsen, Christoph Kamm, Jan C. Koch, Heike Koelbel, Cornelia Koehler, Kirsten Kolzter, Hanns Lochmüller, Albert Ludolph, Alexander Mensch, Gerd Meyer zu Hoerste, Monika Mueller, Wolfgang Mueller-Felber, Christoph Neuwirth, Susanne Petri, Kristina Probst-Schendzielorz, Manuel Pühringer, Robert Steinbach, Ulrike Schara-Schmidt, Mareike Schimmel, Bertold Schrank, Oliver Schwartz, Kurt Schlachter, Annette Schwerin-Nagel, Gudrun Schreiber, Martin Smitka, Raffi Topakian, Regina Trollmann, Matthias Tuerk, Manuela Theophil, Christian Rauscher, Mathias Vorgerd, Maggie C. Walter, Markus Weiler, Claudia Weiss, Ekkehard Wilichowski, Claudia D. Wurster, Gilbert Wunderlich, Daniel Zeller, Andreas Ziegler, Janbernd Kirschner, Astrid Pechmann, SMArtCARE study group

**Affiliations:** 1 Department of Pediatric Neurology and Developmental Medicine and LMU Center for Children With Medical Complexity, Dr. Von Hauner Children’s Hospital, LMU Hospital, Ludwig-Maximilians-University, 80337 Munich, Germany; 2 School of Medicine, Klinikum Rechts Der Isar, Department of Human Genetics, Technical University of Munich, Munich, Germany; 3 Division of Pediatric Neurology, Department of Pediatrics I, Medical University of Innsbruck, Innsbruck, Austria; 4 Department of Children and Adolescents, Ordensklinikum Linz Barmherzige Schwestern, Linz, Austria; 5 Department of Neurology, University Medical Center Schleswig-Holstein, Kiel, Germany; 6 Clinic for Pediatrics, Gesundheitsverbund Wien, Vienna, Austria; 7 School of Medicine, Klinikum Rechts Der Isar, Department of Neurology, Technical University of Munich, Munich, Germany; 8 Department of Neuropaediatrics, Saarland University Hospital, Homburg, Germany; 9 Department of Neuropediatrics, University Hospital Bonn, Center for Pediatrics, Bonn, Germany; 10 Universitätsklinikum Münster Klinik Für Kinder- Und Jugendpädiatrie- Neuropädiatrie, Albert-Schweitzer-Campus 1, Münster, Germany; 11 Department of Neurological Intensive Care and Rehabilitation, Evangelisches Krankenhaus Oldenburg, Oldenburg, Germany; 12 University Hospital Carl Gustav Carus Dresden at Technische Universität Dresden, Dresden, Germany; 13 Department of Child Neurology, Justus-Liebig-University Gießen, Gießen, Germany; 14 Department of Neurology, and Center for Translational Neuro- and Behavioral Sciences (C-TNBS), University Medicine Essen, Essen, Germany; 15 Department for Neuropädiatrie, Landeskrankenhaus Mödling, Mödling, Austria; 16 Department of Paediatric Neurology, University Children’s Hospital Tübingen, Tübingen, Germany; 17 Hannover Medical School, Clinic for Pediatric Kidney-, Liver- and Metabolic Diseases, Hannover, Germany; 18 Department of Pediatrics, University Medical Center Hamburg-Eppendorf, Hamburg, Germany; 19 Department of Neurology, University of Rostock, Rostock, Germany; 20 Klinik Für Neurologie Universitätsmedizin Göttingen, Göttingen, Germany; 21 Department of Pediatric Neurology, Centre for Neuromuscular Disorders, Centre for Translational Neuro- and Behavioral Sciences, University Duisburg-Essen, Essen, Germany; 22 Klinik Für Kinder-Und Jugendmedizin der Ruhr-Universität Bochum Im St. Josef-Hospital, Bochum, Germany; 23 Kliniken Köln, Sozialpädiatrisches Zentrum, Cologne, Germany; 24 Division of Neurology, Department of Medicine, Children’s Hospital of Eastern Ontario Research Institute, The Ottawa Hospital and Brain and Mind Research Institute, University of Ottawa, Ottawa, Canada; 25 Department of Neuropediatrics and Muscle Disorders, Medical Center, Faculty of Medicine, University of Freiburg, Freiburg, Germany; 26 Department for Neurology, University of Ulm, Ulm, Germany; 27 Department of Neurology, University Medicine Halle, Halle, Saale Germany; 28 Department of Neurology, University Hospital Münster, Münster, Germany; 29 Department for Neuropediatrics, University of Wuerzburg, Würzburg, Germany; 30 Neuromuscular Diseases Unit/ALS Clinic, Kantonsspital St. Gallen, St. Gallen, Switzerland; 31 Department of Neurology, Hannover Medical School, Hannover, Germany; 32 Initiative SMA der Deutschen Gesellschaft Für Muskelkranke, Freiburg, Germany; 33 Department of Pediatrics and Adolescent Medicine, Kepler University Hospital Linz, Linz, Austria; 34 Department of Neurology, University Hospital Jena, Jena, Germany; 35 Pediatric Neurology, Pediatrics and Adolescent Medicine, University Medical Center Augsburg, Augsburg, Germany; 36 Department of Neurology, DKD Helios Klinik Wiesbaden, Wiesbaden, Germany; 37 Deutsche Gesellschaft Für Muskelkranke E.V., Freiburg, Germany; 38 Department of Neuropediatrics, Landeskrankenhaus Bregenz, Bregenz, Austria; 39 Medical University Graz Childrens Hospital, Graz, Austria; 40 Department of Neuropediatics, Klinikum Kassel, Kassel, Germany; 41 Department of Neuropediatrics, Medical Faculty Carl Gustav Carus, Technical University Dresden, Dresden, Germany; 42 Department of Neurology, Academic Teaching Hospital Wels-Grieskirchen, Wels, Austria; 43 Department of Pediatrics, Friedrich-Alexander Universität Erlangen-Nürnberg Pediatric Neurology, Erlangen, Germany; 44 Department of Neurology, University Hospital Erlangen, Friedrich-Alexander University Erlangen-Nuremberg, Erlangen, Germany; 45 Centre for Rare Diseases Erlangen (ZSEER), University Hospital Erlangen, Friedrich-Alexander University Erlangen-Nuremberg (FAU), Erlangen, Germany; 46 DRK Kliniken Berlin - Westend Kinderklinik, Berlin, Germany; 47 Department for Neuropediatrics, University of Salzburg, Salzburg, Austria; 48 Department of Neurology, BG-University Hospital Bergmannsheil gGmbH, Heimer Institute for Muscle Research, Ruhr-University Bochum, Bochum, Germany; 49 Friedrich Baur Institute at the Department of Neurology, LMU University Hospital, Ludwig Maximilians University, Munich, Germany; 50 Department of Neurology, Heidelberg University Hospital, Heidelberg, Germany; 51 Charité - University Medicine Berlin, Center for Chronically Sick Children, Berlin, Germany; 52 Department for Neuropediatrics, University Göttingen, Göttingen, Germany; 53 Department of Neurology, Ulm University, Ulm, Germany; 54 German Center for Neurodegenerative Diseases, DZNE, Site Ulm, Ulm, Germany; 55 Faculty of Medicine and University Hospital, Department of Neurology and Center for Rare Diseases, University of Cologne, Cologne, Germany; 56 Department of Neurology, University Hospital Würzburg, Würzburg, Germany; 57 Center for Childhood and Adolescent Medicine, Department of Metabolic Medicine and Pediatric Neurology, University Hospital Heidelberg, Heidelberg, Germany

**Keywords:** Spinal muscular atrophy, SMA, *SMN2*, Age of onset, Neonatal screening, Molecular therapies, Pre-symptomatic treatment

## Abstract

Newborn screening for 5qSMA offers the potential for early, ideally pre-symptomatic, therapeutic intervention. However, limited data exist on the outcomes of individuals with 4 copies of *SMN2*, and there is no consensus within the SMA treatment community regarding early treatment initiation in this subgroup. To provide evidence-based insights into disease progression, we performed a retrospective analysis of 268 patients with 4 copies of *SMN2* from the SMArtCARE registry in Germany, Austria and Switzerland. Inclusion criteria required comprehensive baseline data and diagnosis outside of newborn screening. Only data prior to initiation of disease-modifying treatment were included. The median age at disease onset was 3.0 years, with a mean of 6.4 years. Significantly, 55% of patients experienced symptoms before the age of 36 months. 3% never learned to sit unaided, a further 13% never gained the ability to walk independently and 33% of ambulatory patients lost this ability during the course of the disease. 43% developed scoliosis, 6.3% required non-invasive ventilation and 1.1% required tube feeding. In conclusion, our study, in line with previous observations, highlights the substantial phenotypic heterogeneity in SMA. Importantly, this study provides novel insights: the median age of disease onset in patients with 4 *SMN2* copies typically occurs before school age, and in half of the patients even before the age of three years. These findings support a proactive approach, particularly early treatment initiation, in this subset of SMA patients diagnosed pre-symptomatically. However, it is important to recognize that the register will not include asymptomatic individuals.

## Introduction

Spinal muscular atrophy (SMA) is a genetic disorder characterized by the degeneration of motor neurons in the spinal cord and brain stem, leading to muscle weakness and wasting. The disease is caused by a deficiency of the survival motor neuron (SMN) protein, which is crucial for the normal development and function of motor neurons [1, 2]. SMA is the most common neurodegenerative disease in childhood and the second most common recessive disease after cystic fibrosis, with an incidence ranging from 1:6000 to 1:11,000 [3].

A bi-allelic loss of the *SMN1* gene, often due to a homozygous deletion, is responsible for the autosomal recessive disease in over 95% of cases, resulting in SMN protein deficiency [4]. Approximately 5% of cases are attributed to point mutations in one or both alleles [5]. The *SMN1* gene is responsible for producing most of the functional SMN protein. Humans also possess a gene called *SMN2*, which is paralogous to *SMN1* but differs by only a few nucleotides. As a result, *SMN2* primarily produces non-functional protein due to aberrant splicing during transcription. The severity of SMA, except in cases involving rarer, less understood genetic modifiers [6] is largely determined by the quantity of functional SMN protein generated by the *SMN2* gene. This compensatory mechanism partially mitigates the loss of *SMN1* and modulates the SMA phenotype because a small proportion of *SMN2* transcripts undergo alternative splicing. This results in the production of approximately 5–10% of full-length functional SMN protein, with the severity of SMA strongly influenced by individual variations in *SMN2* copy number.

Three SMN-targeted medications have been approved in Europe and the US between 2016 and 2021: Nusinersen (Spinraza®), onasemnogene abeparvovec xioi (Zolgensma®), and risdiplam (Evrysdi®). These medications either replace the deleted *SMN1* gene using a viral vector or improve aberrant splicing of the *SMN2* gene. All three of these active agents can provide the missing SMN protein [7–11]. However, the timing of treatment is crucial for its effectiveness, as damaged motor neurons poorly regenerate even when SMN protein is restored. Therefore, a substantial number of additional countries worldwide now have SMA included in newborn screening programs in pilot projects or for regular clinical use. It is currently being tested in other countries.

According to the literature, individuals with four copies of *SMN2* typically exhibit a milder form of SMA compared to those with fewer copies [12, 13], and there are cases of individuals who remain mildly symptomatic or asymptomatic into advanced age [14, 15]. However, in the literature, it has been more common to classify SMA by type rather than by copy number, and there is limited available data on populations exclusively composed of individuals with 4 copies of *SMN2*. Existing studies have reported significant variability in the clinical presentation and disease progression of SMA in individuals with 4 *SMN2* copies (16, 17). A recent study published in 2022, which originally focused on gender differences, reported a median age of onset for their 4-copy SMA cohort of 4.75 years (13), and another very recent study from Italy, which focused on disease progression and subgroup classification, revealed that 22% of their patients developed the disease before the age of 3 years [16]. In a recent study from the German SMA Newborn Screening Model Projects, we were able to demonstrate that a notable proportion of SMA patients with 4 *SMN2* copies (5 of 7 patients, all subjects investigated under the age of 5) developed the disease between 1.5 and 4 years, with the majority experiencing very subtle symptoms [17], but some individuals suffering from significant and irreversible motor regression.

Overall, there is no description of the natural history and no consensus on treatment indications for this subset of SMA. This lack of agreement is also evident in the management following newborn screening, which has been introduced in several countries in recent years [18–21]. Following a positive newborn screening for SMA, a decision must be made concerning therapy. There is a clear consensus that individuals with 2 and 3 copies of *SMN2* should receive immediate treatment, while there is uncertainty regarding treatment indications for those with 4 copies of *SMN2* [22]. This disparity is reflected in the 2018 recommendations from the US expert group [23], which recommended a watchful waiting approach for individuals with 4 *SMN2* copies after NBS, and their 2020 guideline revision [24], which suggested early initiation of therapy.

Further research is required to enhance our comprehension of the natural history and clinical progression of SMA with 4 *SMN2* copies, along with identifying factors that could potentially impact disease severity and progression in this patient group. In this manuscript, we present a standardized retrospective evaluation of natural history data drawn from the SMA registry “SMArtCARE” involving 268 patients with 4 *SMN2* copies.

## Materials and methods

SMArtCARE, a disease-specific registry, currently encompasses 58 participating centres in Germany, Austria, and Switzerland. Its primary objective is to collect prospective, longitudinal data on SMA patients. Additionally, it retains retrospective data (medical history) acquired prior to inclusion in the registry and before therapy initiation. As of July 2023, the registry contains information on 1,648 patients of varying ages, SMA types, and treatment modalities. To be eligible for inclusion in SMArtCARE, patients must meet two criteria: they must possess genetically confirmed 5q SMA and provide written informed consent, obtained either from the patient or their caregiver.

Since the implementation of SMArtCARE in 2018, data collection occurs prospectively during routine patient visits to capture real-world outcomes. To maintain consistency, standardized case report forms are utilized to document data rather than extracting information from medical records. These forms adhere to international consensus guidelines for SMA registries and encompass various aspects such as motor function, motor milestones, respiratory and orthopaedic symptoms, adverse events, and genetic test results. Treating physicians record genetic test results, including *SMN2* copy numbers, based on the patients' original genetic test results. Currently, 90.4% of all patients in the registry have undergone *SMN2* copy number determination, with 23.2% possessing 4 *SMN2* copies. It is important to note that *SMN2* copy numbers are not centrally assessed within the SMArtCARE registry, but are entered by the treating clinicians based on the results from the genetic laboratories.

For the analysis of the patient cohort, we exclusively considered patients registered with 4 *SMN2* copies. Inclusion criteria comprised a minimum age of 18 months and either complete attainment of motor milestones (independent walking) before initiating drug therapy or, if independent walking had not been achieved, a minimum age of 5 years at the onset of drug therapy. Patients identified as presymptomatic through newborn screening were excluded from the analysis.

The following parameters were extracted from the registry for statistical analysis: "year of birth," "age at milestones" (sitting unsupported, walking unsupported), "age at symptom onset," "first symptoms or signs leading to suspicion of SMA (free text field)," "wheelchair use," "nutrition (use of feeding tube)," "presence and/or surgery of scoliosis," "need for mechanical ventilation," and the results of the motor tests "6MWT," "RULM," and "HFSME." These baseline characteristics were obtained from the treatment centres through a patient/family medical history survey.

Statistical analyses were conducted using the "R" programming language (Open-Source-Software) and Microsoft Excel 2016 (Microsoft Corporation, Redmond, Washington, USA). The results underwent manual verification for plausibility, and individual patient entries were reconciled. Implausible entries were either corrected if unequivocal or changed to 'unknown'. In the case of 20 patients, the entry "sitting independently was not achieved" was modified to "sitting independently achieved at an unknown age," as this was evidently an incorrect entry, given that all these patients were ambulatory at baseline, corroborated by the "walk test" results. The same correction applies to 18 entries regarding the acquisition of the milestone "walking unaided."

## Results

A total of 303 patients met the criteria of being at least 18 months of age and having 4 copies of *SMN2*. Thirty-two patients, born between 2018 and 2021, were detected by newborn screening and were, therefore, excluded. Three patients, born between 2015 and 2018, initiated drug therapy before achieving independent sitting/walking, leading to their exclusion. Consequently, 268 patients were included. The years of birth ranged from 1948 to 2016, and the patients’ ages at baseline ranged from 3 to 75 years (median 29.6, mean 27 years).

### Age of symptom onset

**Fig. 1:**
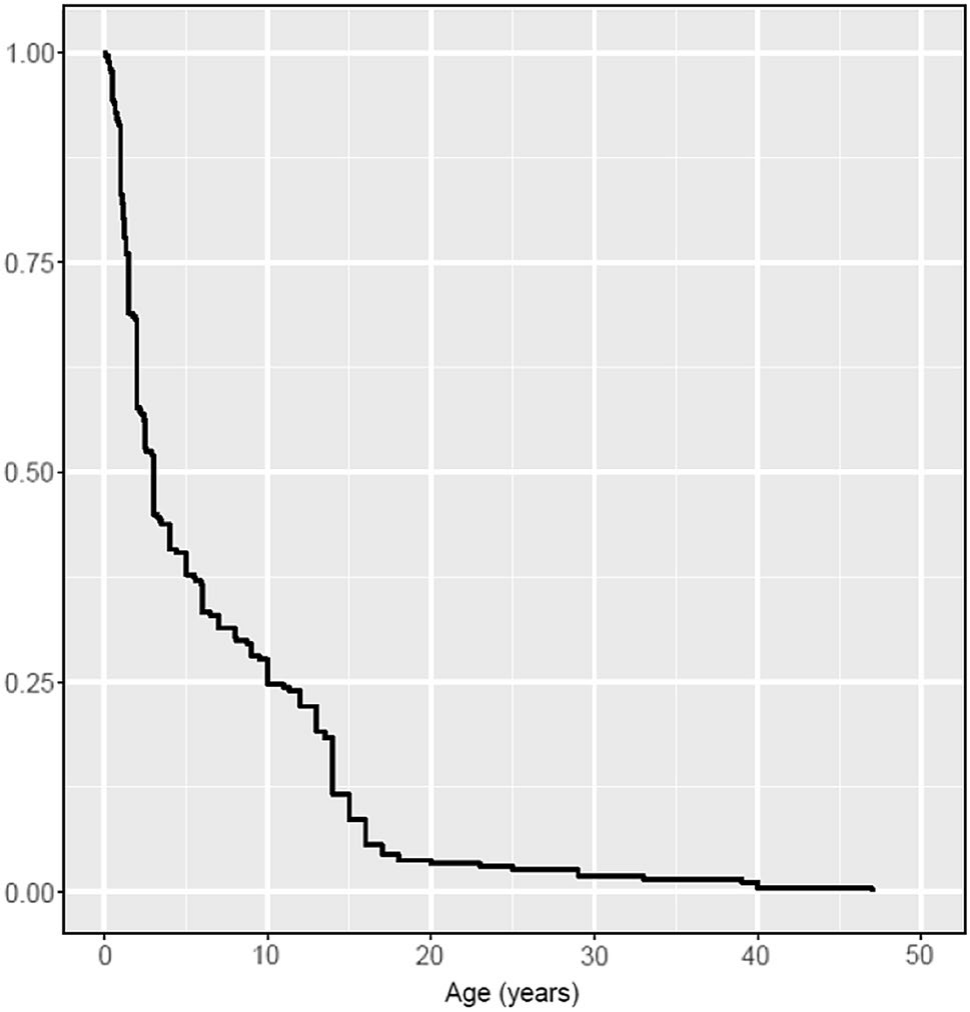
Kaplan–Meier curve for age at disease onset: by the age of 18 years, approximately 95% of patients with four copies of *SMN2* was affected by the disease

Disease onset ranged from 1 month to 47 years of age (median 3.0 years, mean 6.4 years; information was available in all patients). 147 patients (55%) experienced disease onset within the first 36 months of age. The age at symptom onset is presented as a Kaplan–Meier curve in Fig. 1.

### Type of first symptoms

The most common initial symptoms included gait instability, muscle weakness, delayed motor development, frequent falls, decreased performance in sports compared to peers, muscle hypotonia in infancy, tremor, fatigue, and difficulty running and/or climbing stairs. Table 1 provides an overview.

**Table 1:** Overview of patient characteristics related to quality of first symptoms, resulting from free text entries

Symptom	Number of patients
Gait instability	67
Muscle weakness	89
Delayed motor development	34
Frequent falls	21
Muscle hypotonia in infancy	15
Decreased sports performance compared to peers	15
Tremor	14
Fatigue	8
Difficulty running and/or climbing stairs	7
Decline in motor development	7
Stagnation in motor development	6
Muscle pain in the legs	5
Coordination/balance problems	2
Muscle atrophy	1
Known SMA Patient in the Family	1
No speech development	1
Reduced fetal movements during pregnancy	1

Multiple responses were taken into account

### Motor milestones

The cohort learned to sit unaided at ages ranging from 5 to 30 months (median 9.2 months; data were available in 120 patients). Seventeen patients (6.3%) lost the ability to sit unaided at an age of 9 months to 67 years of age (mean 15 years; data were available in 11 patients). Nine patients (3.3%) did not achieve independent sitting.

Independent walking was achieved at ages ranging from 8 months 10.5 years (median 14 months; data were available in 125 patients). Forty-two patients (15.7%) did not attain the ability to walk without support.

### Ambulation

**Fig. 2:**
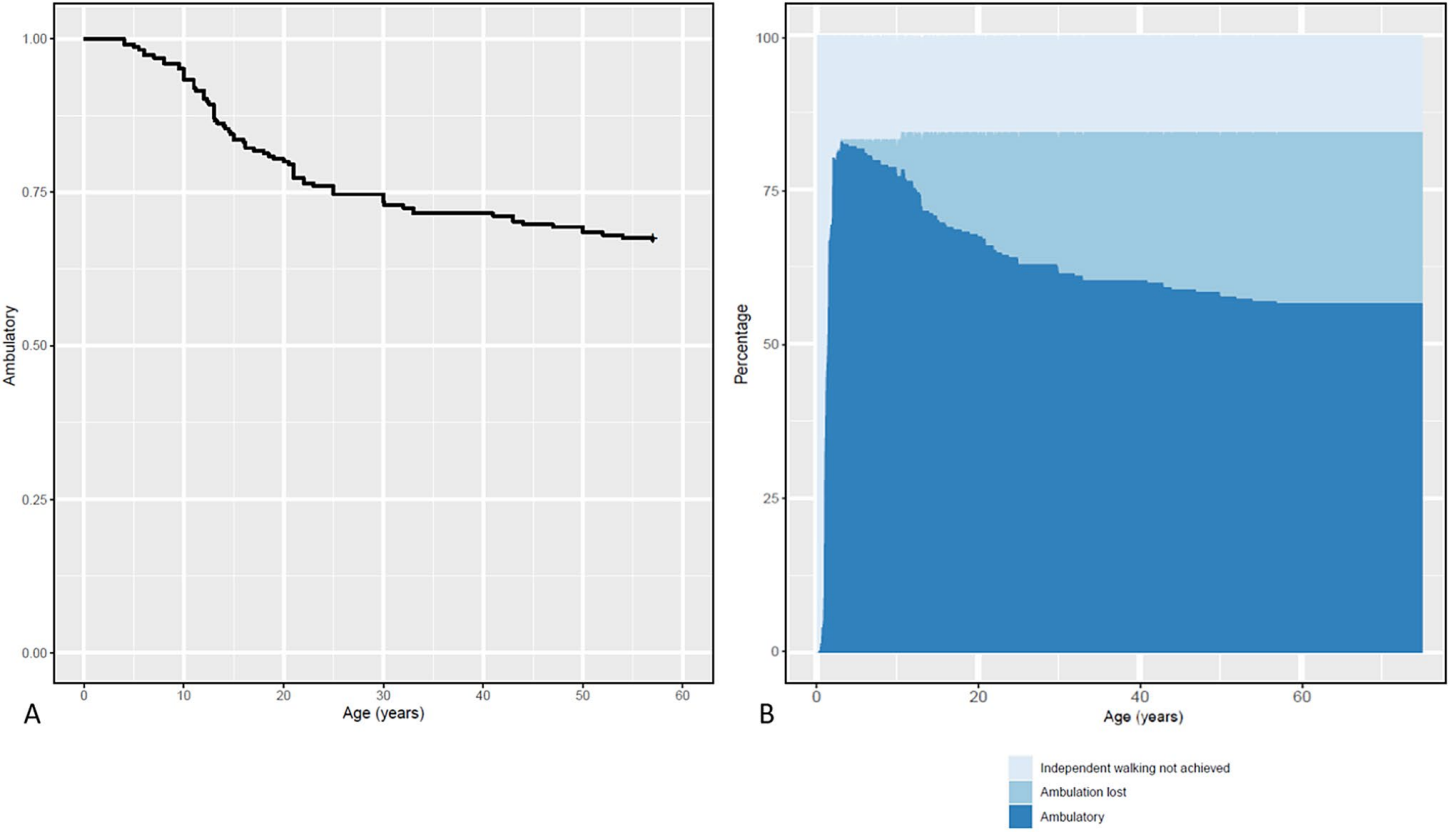
**A** Kaplan–Meier curve for loss of walking ability. By the age of 54, 32.5% of our cohort had lost their ability to walk without assistance. Note: For 20% of patients in this study cohort who lost independent ambulation, the exact age is unknown. Accordingly, 20% of the patients who were still able to walk were subtracted to create this curve. **B** Ambulation as a function of age for the entire cohort. Missing values were estimated from all known values

Out of 226 patients who achieved independent walking, 75 (33.1%) eventually lost this function at ages ranging from 13 months to 57 years (median 21.2 years; information on age at walking loss was available in 62 patients). The age at which the ability to walk was lost is illustrated as a Kaplan–Meier curve in Fig. 2A. Thirty-nine patients (14.5%) used a wheelchair part-time, and full-time wheelchair use was reported by 106 patients (39.5%). Figure 2B displays ambulation as a function of age for the entire cohort, considering those who did not achieve independent walking (upper, pale blue shading). The time between "age at symptom onset" and "loss of ambulation" ranged from 24 months to 51 years, with a median of 17 years.

### Motor scores

**Fig. 3:**
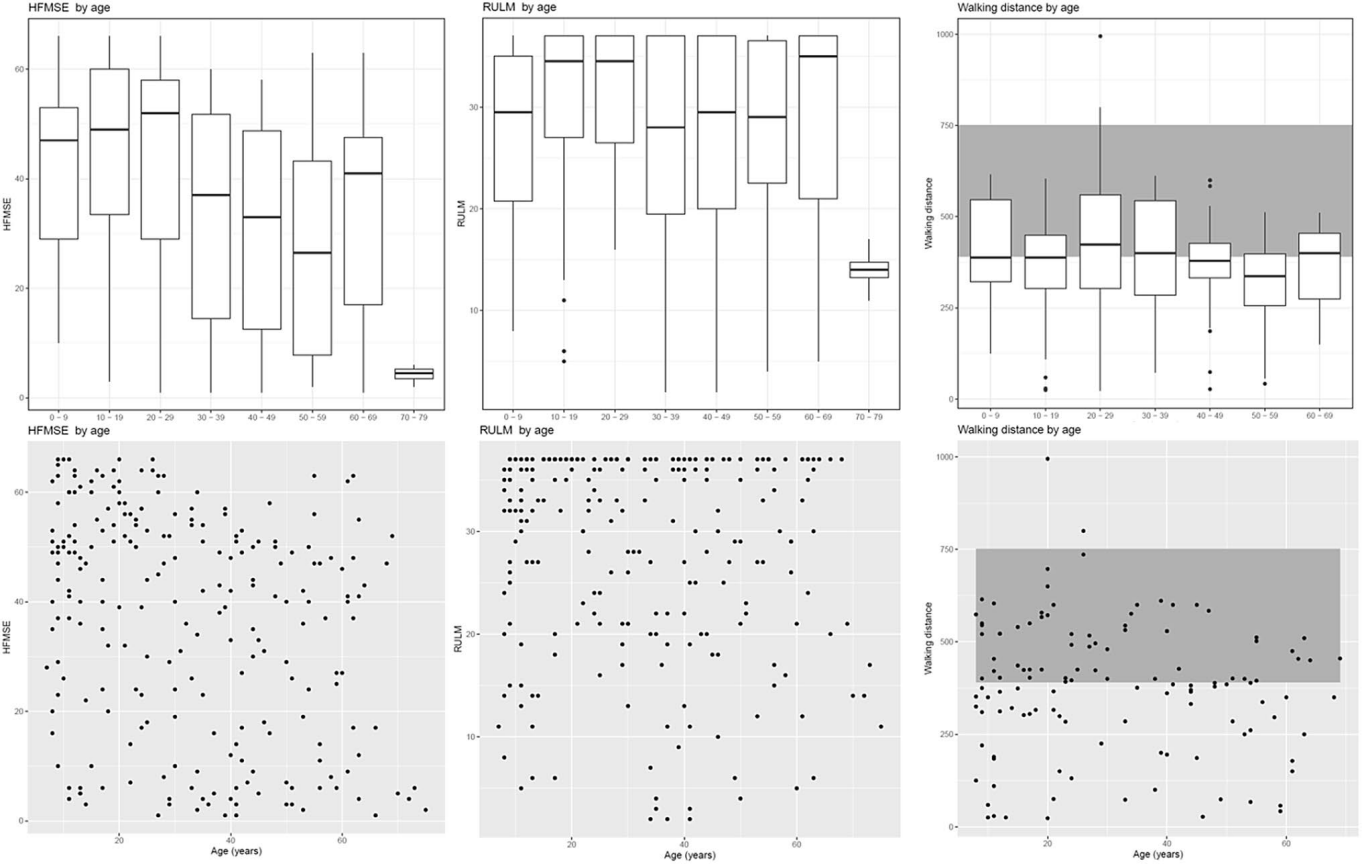
Results of the motor function tests in Baseline. The wide variability of the disease independent of age is evident, with patients ranging from severely to mildly affected in all age groups up to 69 years. It can be assumed for both HFSME and RULM, that full scores are expected from the age of six onwards. Reference values for the 6MWT are shaded in a grey area on the chart

Baseline values for the motor scales "HFSME" (Hammersmith Functional Motor Scale Extended), "RULM" (Revised Upper Limb Assessment), and "6MWT" (6-Minute Walk Test) were available for 230 patients (HFSME), 241 patients (RULM), and 122 patients (6MWT). The results are depicted as scatter plots and box plots (the latter grouped in decades) in Fig. 3. It can be assumed that for both HFSME and RULM, that full scores are expected from the age of six onwards. Reference values for advanced age, where a physiological decline in scores might be expected, do not exist. Reference values for the 6MWT, compiled from a study on healthy populations [25] are shaded in a grey area on the chart.

### Scoliosis/scoliosis surgery

114 patients (42.8%; information available in 266 patients) had scoliosis. Scoliosis surgery had been performed on 28 (10.4%; information available for all patients).

### Correlation of scoliosis (surgery) and age of symptom onset

Patients who underwent scoliosis surgery experienced symptom onset between 1 month and 5.5 years (median 12.5 months). The age at symptom onset for all scoliosis patients had a median of 1.6 years and a mean of 3.6 years. Among the 147 patients with symptom onset within the first 36 months of age, 83 (57%) had scoliosis.

### Tube feeding

Three of the patients (1.1%) utilized a gastric or nasal feeding tube. The age at the initiation of tube feeding was 6.8, 7.3, and 28.9 years, respectively.

### Ventilation

Seventeen patients (6.3%) received non-invasive ventilation. The onset of ventilation ranged from 8 months to 49.8 years (median 19.8 years, mean 23.0 years).

### Correlation between motor function and ventilator use

Among the 17 ventilated patients, five learned to walk independently, but four of them subsequently lost this ability. Additionally, one of them also lost the ability to sit unaided. In contrast, among the 12 ventilated patients who never acquired the ability to walk independently, one also lost the ability to sit unaided. Conversely, among the 42 patients who never achieved independent walking, 30 did not require ventilation.

## Discussion

The natural history of SMA in individuals with four copies of *SMN2* remains less well understood than in other types of SMA. However, there is a widely accepted consensus that emphasizes the paramount importance of early detection and treatment of SMA, regardless of *SMN2* copy number, to improve overall outcomes. In Europe, treatment options for SMA patients with four *SMN2* copies include SMN-targeted therapies such as nusinersen and risdiplam, both of which have been shown in clinical trials to increase SMN protein levels and improve motor function. However, there is a lack of consensus regarding the optimal timing for initiating drug therapy in pre-symptomatic patients with four copies of *SMN2*. In addition, there is an ongoing debate about the potential scenario in which SMA does not manifest until adulthood, potentially avoiding years of unnecessary treatment [22].

The study aims to address the existing ambiguities in determining treatment indications for individuals with higher *SMN2* copy numbers, primarily due to the paucity of long-term data in this context. To address this knowledge gap, we present a robust dataset derived from an extensive cohort of patients with SMA, specifically characterised by the possession of four copies of the *SMN2* gene. We performed a standardised query of the SMA Registry of the D-A-CH Region, which now includes more than 1600 patients. 268 patients with 4 copies of *SMN2* met the inclusion criteria and could be analysed.

Our findings not only support but also refine the prevailing assumption that the phenotypic diversity among individuals with four copies of *SMN2* is remarkably large. In particular, our assessment of motor function at different ages highlights the substantial individual heterogeneity observed in the motor abilities of these patients.

However, perhaps the most important finding of this study is the age of symptom onset (Fig. 1). In our large cohort of symptomatic patients, the median age of onset was only 3 years. In more than half of the cohort, the first symptoms occurred before the age of 3 years. By the age of 18, about 95% of patients with four copies of *SMN2* were affected by the disease. Of course, this number must be treated with caution, as people with very little or no symptoms would not be included in the registry. Nevertheless, these data support the proposal of the Independent Expert Commission 2020 [24] to discuss the timing of drug treatment after pre-symptomatic diagnosis. Our data suggest that in the majority of patients with 4 copies of *SMN2*, the amount of SMN protein is insufficient to prevent motor neuron damage in the long term. As SMN production is highest in early life [26], early initiation of therapy in infancy may be appropriate to prevent motor neuron death if the diagnosis is made at a pre-symptomatic stage [24].

This is supported by the fact that in terms of loss of ambulation, as shown in Fig. 2, almost a third of the cohort lost their previously achieved ability to walk independently. Looking at the age at which this loss of ambulation occurred, there was, as expected, a wide age range with a distribution that is relatively uniform. This observation largely confirms the findings documented in the comprehensive review by Wirth et al. in 2021 [27] and the recent study by Ricci et al., who found an overall risk of walking loss of 35% in their cohort of SMA types 3 and 4 [16]. The time between "age at symptom onset" and "loss of ambulation" also showed a large variation.

In terms of ventilatory support, the prevalence of patients requiring non-invasive ventilation among those with four *SMN2* copies was relatively small. Of course, the absence of ventilation does not exclude respiratory muscle involvement (data on vital capacity were not available at baseline), but this suggests that the disease course associated with four copies of *SMN2* may have a comparatively milder effect on the respiratory musculature compared to patients with a lower copy number. However, an additional explanation for this "lower respiratory prevalence" could be due to the gradual progression of symptoms, with patients adapting their daily activities and not actively reporting respiratory problems. It may also be due to increased "medical awareness" within the healthcare community, which is now more vigilant than in previous years in monitoring and treating any co-morbidities associated with SMA [28].

Scoliosis was present in 42% of our patients, whereas the prevalence of scoliosis in the otherwise healthy population is only about 2–4%. However, only one quarter of scoliosis patients required spinal surgery. As almost all patients with SMA type 2 develop scoliosis in early childhood [29], we correlated the age of symptom onset in our patient cohort with the presence of scoliosis. Indeed, in patients who underwent scoliosis surgery, symptoms appeared between the ages of 1 month and 5.5 years. Looking at the age at symptom onset for all patients with scoliosis, the median/mean is 1.6/3.6 years, which is significantly lower than in the overall cohort. It is therefore plausible that early onset SMA with 4 *SMN2* copies is associated with an increased risk of severe scoliosis. To investigate this further, we looked at the subgroup with disease onset up to 36 months of age. Among them, 56% had scoliosis, which was higher than the overall rate of 43%, but not as high as in the type 2 population. In addition, the proportion of patients in our cohort who did not achieve independent walking exceeded the known range for SMA type 2 patients with 4 *SMN2* copies [30]. It can therefore be concluded that the "old classification", which has gradually been abandoned and is now more often referred to as "sitter" or "walker", does not accurately represent the types of SMA and that there is a certain continuum between type 2 and type 3, even among 4 *SMN2* copies.

Our clinical data reflect the severe consequences of SMN deficiency in SMA even with 4 *SMN2* copies. The overall picture is one of an aggressive disease with an early onset and a high number of wheelchair-bound patients, with a very broad phenotypic spectrum. This broad spectrum, which is also known in SMA with 3 *SMN2* copies, is still poorly understood. One reason may be that there is no guarantee that all the *SMN2* copies found are able to produce SMN protein. Certainly, siblings with different SMA phenotypes and identical *SMN2* copy number and markers have been described, suggesting that the genetic background around the SMA locus alone is not sufficient to explain the phenotypic variability [31, 32]. However, it is possible that one or more copies are defective [33]. Several *SMN2* variants have been identified that are associated with milder phenotypes [34–37], but less is known about *SMN2* variants that lead to more severe SMA phenotypes or whether certain *SMN2* variants are associated with a weaker response to mRNA treatment. Sequential analysis of individual *SMN2* copies is not yet established in routine diagnostics and, unlike other potential genetic modifiers, has not been sufficiently studied to determine its validity.

However, individual patients with 4 copies of *SMN2* who remained asymptomatic over a long period of life have been published. There has been no significant increase in similar reports over the years, suggesting that this phenomenon may be rare. Unfortunately, there are no known modifiers that can be routinely tested to predict progression, which makes counselling families with SMA and 4 *SMN2* copies diagnosed through a newborn screening program very difficult. In general, the value of electrophysiology, such as EMG to detect denervation/re-innervation, or the Motor Unit Number Index MUNIX, which has been shown to be feasible in children after the age of 5 in proximal muscles in older children [38, 39], can be helpful in making treatment decisions. However, these are invasive procedures that are increasingly unavailable in paediatric treatment centres and are, therefore, more theoretical (also reflected in the fact that almost no electrophysiological data from our patient cohort are recorded in the registry). Of course, therapy indication must be weighed against the potential risks (e.g. long-term intrathecal therapy or the short observation period of oral small-molecule therapy). But given that once damaged, motor neurons do not regenerate and therefore any form of SMA should be treated strictly pre-symptomatically, the data from this study support a proactive approach, despite uncertainty about the expected course of the disease.

Our data also support the value of detecting SMA with 4 *SMN2* copies in newborn screening. The goal of newborn screening is to diagnose patients at a pre-symptomatic stage, before the onset of motor neuron disease. Failure to report pathological findings in children with 4 copies puts patients at risk of developing severe forms of SMA. Therefore, the results of our studies call for surveillance programmes of asymptomatic gene carriers aimed at detecting early signs of disease, which may include neurochemical markers.

## Limitations

There is a potential bias in the determination of the *SMN2* copy number, as we know that especially the older determinations of the *SMN2* copy number can be very inaccurate. It is therefore possible that not all patients have the correct copy number and that some patients with three copies may have been included, which was shown in the recent work by Ricci et al. to be 10% in their 4 *SMN2* copy cohort [16]. However, the observation that only 6.3% are ventilator dependent suggests that the substantial subset of patients with early motor symptoms from infancy have not been inadvertently included due to an incorrect copy number.

Another limitation is to consider is that individuals with minimal or no symptoms may not have received a diagnosis, potentially resulting in their under-representation in the registry. However, this limitation is mitigated by the fact that the overall prevalence of 4 *SMN2* copies in the registry is currently 23%, which is consistent with recent incidences reported in new-born screening projects that do not miss cases of homozygous *SMN1* deletion [40].

In Fig. 2B, it has not been taken into account that the observation period varies from patient to patient. The youngest patients are only seven years old, so some will lose their ability to walk in the future—this is not reflected in the graph.

## Conclusion

The early onset of symptoms in our cohort, coupled with the expected wide clinical variability, strongly supports the characterization of SMA in individuals with four *SMN2* copies as primarily a childhood disease. In particular, more than half of our cohort had their first symptoms before the age of 36 months and almost 95% of patients were affected by the disease by the age of 18 years. These findings must be taken into account when considering the feasibility of a watchful waiting approach to SMA therapy. Our data strongly support a proactive stance involving early initiation of treatment in this subset of SMA patients, particularly if a pre-symptomatic diagnosis is made.

## Data Availability

Detailed data are available from the SMArtCARE registry or from the corresponding author’s institutions, upon reasonable request.

## Abbreviations

SMA: Spinal muscular atrophy
SMN: Survival motor neuron
6MWT: Six-minute walk test
RULM: Revised upper limb module
HFSME: Hammersmith functional motor scale expanded

## References

[1] Burghes AH, Beattie CE (2009). Spinal muscular atrophy: why do low levels of survival motor neuron protein make motor neurons sick?. Nat Rev Neurosci.

[2] Pellizzoni L, Yong J, Dreyfuss G (2002). Essential role for the SMN complex in the specificity of snRNP assembly. Science.

[3] Verhaart IEC (2017). Prevalence, incidence and carrier frequency of 5q-linked spinal muscular atrophy - a literature review. Orphanet J Rare Dis.

[4] Lefebvre S (1995). Identification and characterization of a spinal muscular atrophy-determining gene. Cell.

[5] Lorson CL (1999). A single nucleotide in the SMN gene regulates splicing and is responsible for spinal muscular atrophy. Proc Natl Acad Sci U S A.

[6] Wirth B, Garbes L, Riessland M (2013). How genetic modifiers influence the phenotype of spinal muscular atrophy and suggest future therapeutic approaches. Curr Opin Genet Dev.

[7] Finkel RS (2017). Treatment of infantile-onset spinal muscular atrophy with nusinersen: a phase 2, open-label, dose-escalation study. Lancet.

[8] Mercuri E (2018). Nusinersen versus sham control in later-onset spinal muscular atrophy. N Engl J Med.

[9] Mendell JR (2017). Single-Dose gene-replacement therapy for spinal muscular atrophy. N Engl J Med.

[10] Strauss KA (2022). Onasemnogene abeparvovec for presymptomatic infants with three copies of SMN2 at risk for spinal muscular atrophy: the Phase III SPR1NT trial. Nat Med.

[11] Darras BT (2021). Risdiplam-treated infants with type 1 spinal muscular atrophy versus historical controls. N Engl J Med.

[12] Pechmann A, Kirschner J (2017). Diagnosis and new treatment avenues in spinal muscular atrophy. Neuropediatrics.

[13] Maggi L (2022). Adults with spinal muscular atrophy: a large-scale natural history study shows gender effect on disease. J Neurol Neurosurg Psychiatry.

[14] Jedrzejowska M (2008). Unaffected patients with a homozygous absence of the SMN1 gene. Eur J Hum Genet.

[15] Wirth B (2006). Mildly affected patients with spinal muscular atrophy are partially protected by an increased SMN2 copy number. Hum Genet.

[16] Ricci M (2023). Clinical phenotype of pediatric and adult patients with spinal muscular atrophy with four smn2 copies: are they really all stable?. Ann Neurol.

[17] Blaschek A (2022). Newborn screening for SMA - can a wait-and-see strategy be responsibly justified in patients with four SMN2 copies?. J Neuromuscul Dis.

[18] Kariyawasam DST (2020). The implementation of newborn screening for spinal muscular atrophy: the Australian experience. Genet Med.

[19] Kraszewski JN (2018). Pilot study of population-based newborn screening for spinal muscular atrophy in New York state. Genet Med.

[20] Gailite L (2022). New-born screening for spinal muscular atrophy: results of a latvian pilot study. Int J Neonatal Screen.

[21] Muller-Felber, W., et al., *Newbornscreening SMA - From Pilot Project to Nationwide Screening in Germany.* J Neuromuscul Dis, 2022. 10.3233/JND-221577 PMC9881029 36463459

[22] Muller-Felber W (2020). Infants diagnosed with spinal muscular atrophy and 4 SMN2 copies through newborn screening - opportunity or burden?. J Neuromuscul Dis.

[23] Glascock J (2018). Treatment algorithm for infants diagnosed with spinal muscular atrophy through newborn screening. J Neuromuscul Dis.

[24] Glascock J (2020). Revised recommendations for the treatment of infants diagnosed with spinal muscular atrophy via newborn screening who have 4 copies of SMN2. J Neuromuscul Dis.

[25] Casanova C (2011). The 6-min walk distance in healthy subjects: reference standards from seven countries. Eur Respir J.

[26] Ramos DM (2019). Age-dependent SMN expression in disease-relevant tissue and implications for SMA treatment. J Clin Invest.

[27] Wirth B (2021). Spinal muscular atrophy: in the challenge lies a solution. Trends Neurosci.

[28] Leibrock B (2023). Areas of improvement in the medical care of SMA: evidence from a nationwide patient registry in Germany. Orphanet J Rare Dis.

[29] Mullender M (2008). A Dutch guideline for the treatment of scoliosis in neuromuscular disorders. Scoliosis.

[30] Calucho M (2018). Correlation between SMA type and SMN2 copy number revisited: an analysis of 625 unrelated Spanish patients and a compilation of 2834 reported cases. Neuromuscul Disord.

[31] Cusco I (2006). SMN2 copy number predicts acute or chronic spinal muscular atrophy but does not account for intrafamilial variability in siblings. J Neurol.

[32] Jones CC (2020). Spinal muscular atrophy (SMA) subtype concordance in siblings: findings from the cure SMA cohort. J Neuromuscul Dis.

[33] Chen X (2023). Comprehensive SMN1 and SMN2 profiling for spinal muscular atrophy analysis using long-read PacBio HiFi sequencing. Am J Hum Genet.

[34] Prior TW (2009). A positive modifier of spinal muscular atrophy in the SMN2 gene. Am J Hum Genet.

[35] Vezain M (2010). A rare SMN2 variant in a previously unrecognized composite splicing regulatory element induces exon 7 inclusion and reduces the clinical severity of spinal muscular atrophy. Hum Mutat.

[36] Wu X (2017). A-44G transition in SMN2 intron 6 protects patients with spinal muscular atrophy. Hum Mol Genet.

[37] Ruhno C (2019). Complete sequencing of the SMN2 gene in SMA patients detects SMN gene deletion junctions and variants in SMN2 that modify the SMA phenotype. Hum Genet.

[38] Neuwirth C, Weber M (2020). MUNIX in children with spinal muscular atrophy: an unexpected journey. Muscle Nerve.

[39] Mendonca RH (2021). Motor unit number index (MUNIX) in children and adults with 5q-spinal muscular atrophy: variability and clinical correlations. Neuromuscul Disord.

[40] Vill K (2021). Newborn screening for spinal muscular atrophy in Germany: clinical results after 2 years. Orphanet J Rare Dis.

